# Genomic Imprinting in Animals: Evolutionary Conservation with Adaptive Flexibility

**DOI:** 10.3390/cells15181709

**Published:** 2026-09-20

**Authors:** Stepan N. Belyakin, Prim B. Singh

**Affiliations:** 1 Epigenetics Laboratory, Department of Natural Sciences, Novosibirsk State University, Pirogov Str. 2, 630090 Novosibirsk, Russia; 2 Department of Biomedical Sciences, School of Medicine, Nazarbayev University, Kerey Zhanibek Khandar Street 5/1, Astana 010000, Kazakhstan

**Keywords:** parent-of-origin effects, mammals, marsupials, insects, worms, imprinting

## Abstract

Imprinting is an epigenetic mechanism found in many diploid organisms that enables discrimination between genetic material inherited from one or the other parent. It has been predominantly studied in mammals, where, in the adult, it affects the expression of a limited set of genes (in most higher mammals) or leads to paternal X chromosome inactivation in marsupials. DNA CpG methylation and heterochromatic epigenetic marks play a crucial role in these processes. Imprinting also exists in other organisms, where it can cause unusual parent-of-origin-dependent effects. Two classical examples are whole paternal genome inactivation in mealybug males and selective paternal X chromosome elimination in fungus gnats. Although these manifestations of imprinting are quite exotic, they share features in common with those observed in mammals, implying that, despite the differences, the core mechanism, the H3K9me3:HP1:H4K20me3 pathway, is evolutionarily conserved across animal phyla. Here, we discuss recent advances that provide further evidence supporting this concept.

## 1. Introduction

The term imprinting refers to mechanisms that distinguish the parental origin of genetic material. The term is attributed to Helen Crouse [1], who used it to describe the unusual behavior of X chromosomes in *Sciara coprophila* (later renamed *Bradisia coprophila*), a fungus gnat. It was consequently applied to various epigenetic mechanisms that discriminate the parental origin of genes, chromosomes, or whole chromosome sets in different taxa, from nematodes to mammals, manifesting in diverse and sometimes bizarre ways [1,2,3,4,5,6,7]. Currently, the term genomic imprinting has become mammal-centric, while examples from other animals are considered anecdotal.

Nevertheless, existing data suggest that some molecular components and fundamental principles of imprinting are conserved, while serving different biological needs in different organisms. Rather than providing a comprehensive catalogue of imprinting phenomena, this review focuses on these conserved components across highly divergent animal taxa. By comparing these systems, we propose that conserved heterochromatin mechanisms can represent an evolutionarily reusable molecular module that has been repeatedly adapted to distinguish parental genomes and generate diverse parent-of-origin effects. This perspective highlights important questions about how conserved epigenetic mechanisms contribute to imprinting in different biological contexts, giving rise to diverse forms and functions of parent-of-origin effects across animal taxa.

## 2. Imprinting in Mammals

In mammals, imprinting manifests as selective activity of certain genes depending on their parental origin. In placental mammals, the number of such genes is small—there are roughly 200 imprinted genes in mice and humans [8]. In marsupials, imprinting regulates the dosage compensation of the X chromosome.

### 2.1. Imprinting in Mice (Placental Mammals)

In mice, normal development requires both maternal and paternal genetic complements, as demonstrated by uniparental disomies [9] and pronuclear transfer experiments [10,11]. These imprinted genes constitute the barrier to parthenogenetic development observed in some other vertebrates, but not in mammals [12]. Approximately 150–260 genes exhibit a parent-of-origin effect in the mouse [8,13].

DNA CpG methylation is an important epigenetic mark involved in gene regulation and imprinting in many organisms [14,15,16]. In mammalian somatic cells, 5 mC occurs at the majority (~70–80%) of CpG dinucleotides [17]. DNA methylation of gene promoters and first exons is associated with transcriptional repression [18]. Within the predominantly methylated mammalian genome, small (~1 kb) CpG-dense regions known as CpG islands (CGIs) are typically associated with gene promoters and generally remain unmethylated, irrespective of gene expression status [19,20]. However, CGIs may acquire methylation under specific conditions, for example during X-chromosome inactivation in females [21]. Furthermore, CGIs are heavily methylated at silenced imprinted loci [15,22].

**Figure 1:**
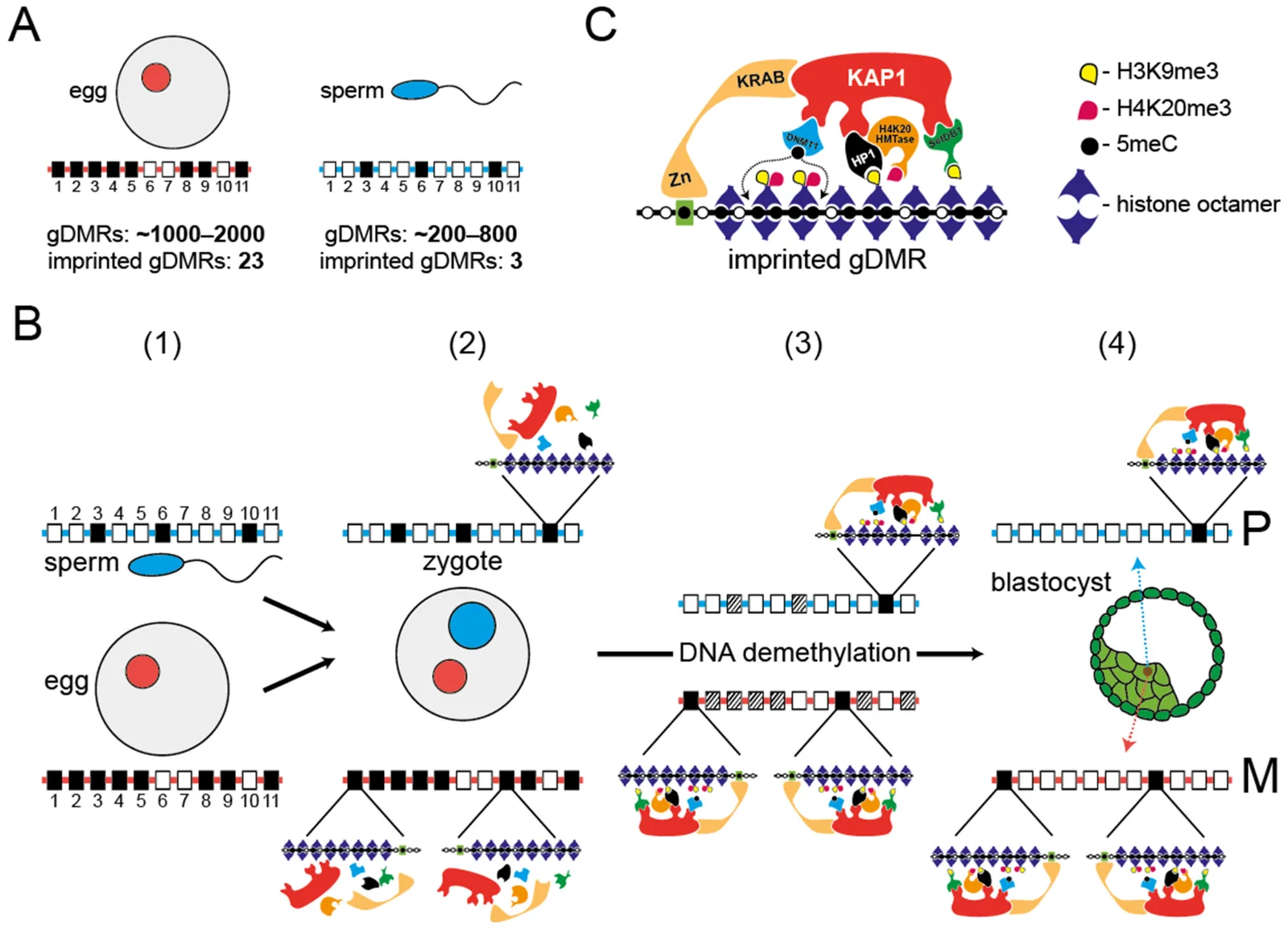
**Maintenance of DNA methylation at imprinted gDMRs through the assembly of a localized heterochromatin-like complex.** (**A**) Schematic methylation pattern and approximate numbers of gDMRs in maternal (red) and paternal (blue) homologous chromosomes. Open rectangles denote non-methylated CGIs, whereas filled rectangles represent methylated CGIs. CGI number 3 is methylated in both parental genomes and thus illustrates the existence of non-differentially methylated regions. Only few of the gDMRs correspond to the imprinted loci. (**B**) Maintenance of DNA methylation at imprinted gDMRs during early embryogenesis. During preimplantation development, most germline methylation marks are erased, but the methylation at imprinted gDMRs is selectively protected through the assembly of localized heterochromatin-like complexes. Non-imprinted gDMRs become progressively demethylated (hatched rectangles). By the blastocyst stage, global DNA methylation reaches its minimum level, while imprinted gDMRs remain methylated. P, paternal homologue; M, maternal homologue. (**C**) Proposed organization of the heterochromatin-like complex assembled at imprinted gDMRs. ZFP57 binds methylated TGCCGC motifs and recruits the co-repressor KAP1, which serves as a scaffold for the recruitment of SETDB1, HP1, DNMT1, and associated chromatin-modifying factors. SETDB1 catalyzes H3K9 trimethylation, which is recognized by HP1. HP1 subsequently promotes H4K20 trimethylation through recruitment of H4K20 histone methyltransferase, completing the H3K9me3:HP1:H4K20me3 interface. Together with DNMT1, this complex maintains DNA methylation at imprinted gDMRs during DNA replication. Adapted from [46].

DNA methylation in higher mammals exhibits complex dynamics during the life cycle [23]. Genome-wide DNA methylation, including at CGIs, is reduced to very low levels in primordial germ cells (PGCs) by the time they migrate to the genital ridge (in mouse, embryonic day 10.5) [24]. At the onset of gametogenesis, the de novo DNA methylation machinery methylates a large number of CGIs, irrespective of whether they are associated with imprinted genes. Different studies estimate that 1000–2000 CGIs (Figure 1A) are subject to de novo methylation by the DNMT3A–DNMT3L complex in oocytes [25,26,27,28,29]. In general, the maternal genome exhibits approximately 40% global CpG methylation [30].

In the male gonad, both DNMT3A and DNMT3B contribute to de novo methylation in combination with DNMT3L [31]. De novo DNA methylation begins in resting prospermatogonia, and methylation patterns are fully established before birth [32,33]. These methylation patterns are faithfully maintained through multiple rounds of cell division prior to the formation of mature sperm. The number of CGIs methylated in sperm has been reported to vary depending on the study, with one identifying 185 methylated CGIs (Smallwood et al., 2011) and another 818 [28] (Figure 1A). In general, sperm DNA is heavily methylated, exhibiting 80–90% overall CpG methylation [34]. Also, in sperm, the genome is compacted with protamines, which are replaced by maternally supplied histones after fertilization to reconstitute the chromatin environment required for proper gene function [35,36].

Approximately 1500 CGIs are differentially methylated between the oocyte and sperm [27,28]. These CGIs are referred to as germline differentially methylated regions (gDMRs). Notably, only ~25 of them are known to be associated with imprinted genes that maintain allele-specific methylation during early development [37,38]. Of these ~25 imprinted gDMRs, only seven have been demonstrated to function as ICRs (Imprinting Control Regions), defined as regions required for parent-of-origin–specific expression [4,15,39]. Thus, with respect to imprinting, de novo DNA methylation of CGIs during murine gametogenesis appears excessive and does not confer specificity to the final methylation pattern.

After fertilization, the paternal and maternal genomes undergo extensive demethylation (Figure 1B), but follow distinct kinetics and mechanisms [40,41,42]. The paternal genome experiences a rapid loss of DNA methylation that is nearly complete by the onset of DNA replication at pronuclear stage 3 [40,41]. In contrast, the maternal genome undergoes predominantly passive, replication-dependent demethylation due to reduced DNMT1 activity [43,44]. DNA demethylation continues during pre-implantation development, reaching its lowest levels at the blastocyst stage, after which de novo methylation is re-established in a lineage-specific manner [45] (Figure 1B).

In contrast, methylation at the ~25 gDMRs of imprinted genes is preserved throughout the demethylation phase by the limited DNMT1 that remains in the nuclei of early embryos [47,48] (Figure 1B). These regions are also protected from the activity of demethylating TET dioxygenases [49,50]. Together, these mechanisms maintain methylation at imprinted gDMRs in the mouse embryo while the rest of the genome undergoes demethylation. This mechanism is essential for imprinting as it confers specificity.

A key maternally provided, sequence-specific factor that preserves imprinted gDMRs from loss of DNA methylation is ZFP57, a member of the KRAB-ZFP family. Loss of maternal and zygotic ZFP57 results in demethylation of multiple gDMRs [51], and deletion of *ZFP57* in ES cells produces a similar effect [52,53]. Allele-specific binding of ZFP57 occurs via a hexamer motif (TGCCGC) present in gDMRs of imprinted genes; ZFP57 exhibits the highest affinity for this motif when the central CpG dinucleotide is fully methylated [52,53,54] (Figure 1C).

ZFP57 nucleates a heterochromatin complex (Figure 1C). Genome-wide studies in ES cells have shown that ZFP57 binding at the methylated allele of gDMRs overlaps with its co-repressor KAP1, the SETDB1 histone methyltransferase, and peaks of H3K9me3 and H4K20me3 [52,55]. ChIP analysis in ES cells demonstrated that HP1γ is recruited to gDMRs by the ZFP57–KAP1 complex [52]. Finally, KAP1 recruits the limited DNMT1 present in early embryos, thereby maintaining DNA methylation at imprinted gDMRs [49]. Protection of methylation at imprinted gDMRs from TET dioxygenase activity is also likely mediated by the SETDB1-dependent heterochromatin complex [56].

Thus, differential DNA CpG methylation at imprinted genes, together with ZFP57 binding, leads to the formation of a heterochromatin complex that protects imprinted loci from demethylation during early embryogenesis and ensures parent-of-origin-specific expression and propagation of the imprinted state through cell divisions. One direction for future research could include a systematic elucidation of the roles of the three homologous mammalian HP1 proteins and their interplay in the establishment and maintenance of DNA methylation at imprinted loci using conditional or constitutive mutations of HP1-encoding genes.

In many imprinted loci, this mechanism is complemented by the involvement of non-coding RNAs (ncRNAs) [15,57]. DNA methylation at the locus control region (LCR) directly regulates the expression of an antisense ncRNA, which in turn controls the expression of the protein-coding genes within the imprinted locus. In these cases, the imprinted LCR functions as the promoter of the ncRNA. On the 5 mC-methylated allele, this promoter is inactive, the ncRNA is not expressed, and the protein-coding genes remain active. Conversely, the unmethylated LCR drives ncRNA expression, leading to repression of the locus [58].

An example of this regulatory logic is the *Igf2r* imprinted cluster, where the paternally expressed non-coding RNA *Airn* is required for *cis*-silencing of the protein-coding genes [59]. Furthermore, the maternally expressed non-coding RNA *H19* from the *Igf2* imprinted cluster acts as a co-repressor at other imprinted loci, including *Slc38a4* and *Peg1*, by recruiting repressors to their methylated LCRs [60]. Thus, in a number of cases, the non-coding RNA is the primary target of imprinting and, in turn, mediates the downstream parent-of-origin effects.

### 2.2. Imprinted X Chromosome Inactivation in Marsupials

In mammals, X chromosome inactivation in females serves as a mechanism of dosage compensation [61]. Males possess a single X chromosome, whereas females have two. To balance the expression of X-linked genes, one X chromosome is silenced in all somatic cells of females [62]. In higher mammals, the X chromosome to be inactivated is chosen randomly in the adult. In marsupials, this process involves imprinting—the X chromosome that is inactivated is invariably paternally-derived [63,64].

**Figure 2:**
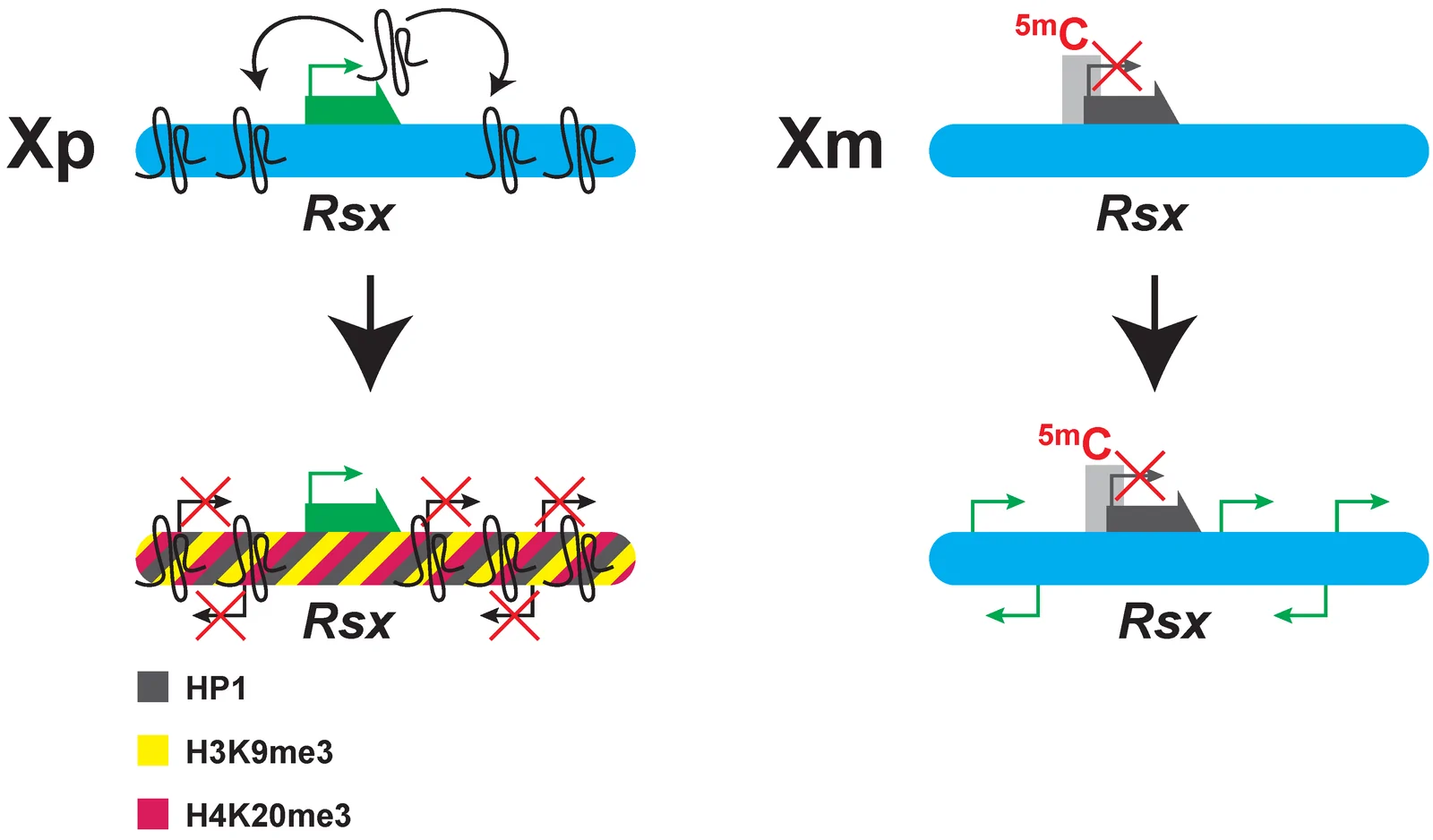
**Imprinted inactivation of the paternal X chromosome during dosage compensation in marsupials.** In females, the *Rsx* non-coding RNA is selectively expressed from the paternal X chromosome. It spreads along the chromosome *in cis* and nucleates the formation of H3K9me3:HP1 heterochromatin, leading to X chromosome inactivation. In the maternal X chromosome, the promoter of the *Rsx* gene is pre-methylated, preventing expression of the ncRNA and thereby leaving the chromosome active.

Similarly to *Xist* RNA in placental mammals (for review see [62,65,66,67]), X chromosome dosage compensation in marsupials involves the non-coding RNA *Rsx*, which is expressed from the paternal X chromosome (Figure 2). *Rsx* RNA accumulates on the X chromosome during its inactivation [62,68]. The process of dosage compensation ultimately results in the establishment of repressive chromatin characterized by H3K9me3, H4K20me3, and HP1 proteins [69,70] (Figure 2). This set of epigenetic markers differs from that observed in *Xist*-dependent X inactivation [70], but overlaps with chromatin components present at imprinted loci in placental mammals (see above).

Interestingly, the *Rsx* transgene inserted into an autosome of murine embryonic stem cells is transcribed and spreads *in* *cis* along the chromosome. Moreover, this coincides with the inactivation of genes on that chromosome, suggesting that *Rsx* indeed plays a central role in dosage compensation in marsupials and providing an attractive model system for studying the evolutionarily conserved components of this mechanism [68].

Notably, in marsupials, DNA of the inactivated paternal X chromosome is generally hypomethylated compared to autosomes and, unexpectedly, to the active X chromosome in males [71,72,73]. However, CGIs in the promoter of the *Rsx* non-coding RNA gene display striking maternal-specific DNA methylation (Figure 2). This methylation pattern is clearly observed on the single X chromosome (of maternal origin) in male tissues and is specifically maintained on the maternal X chromosome in female somatic tissues [71,72]. Both X chromosomes are active in female germ line cells, and the *Rsx* gene is silenced on both [68]. The precise role of methylation in the promoter region of the active *Rsx* allele remains unclear but taken together, these data suggest that the *Rsx* gene represents an imprinted locus on the marsupial X chromosome that ensures selective expression from the paternal chromosome. Altogether, these observations resemble the role of non-coding RNAs in the parent-of-origin effects observed at many imprinted loci in mice, as discussed above. However, in marsupials, imprinted inactivation affects the entire chromosome rather than just a few adjacent genes.

Thus, existing evidence indicates that DNA methylation and epigenetic marks characteristic of constitutive heterochromatin (H3K9me3, H4K20me3, and HP1 protein), as well as non-coding RNA, are involved in imprinted X-chromosome dosage compensation in marsupials, making it mechanistically similar to imprinted loci in placental mammals. Important questions remain unresolved. For example, what molecular mechanism underlies the DNA methylation-dependent regulation of *Rsx* gene activity, and how is this methylation maintained in female tissues?

## 3. Imprinting in Insects

### 3.1. Planococcus citri (Mealybugs)

**Figure 3:**
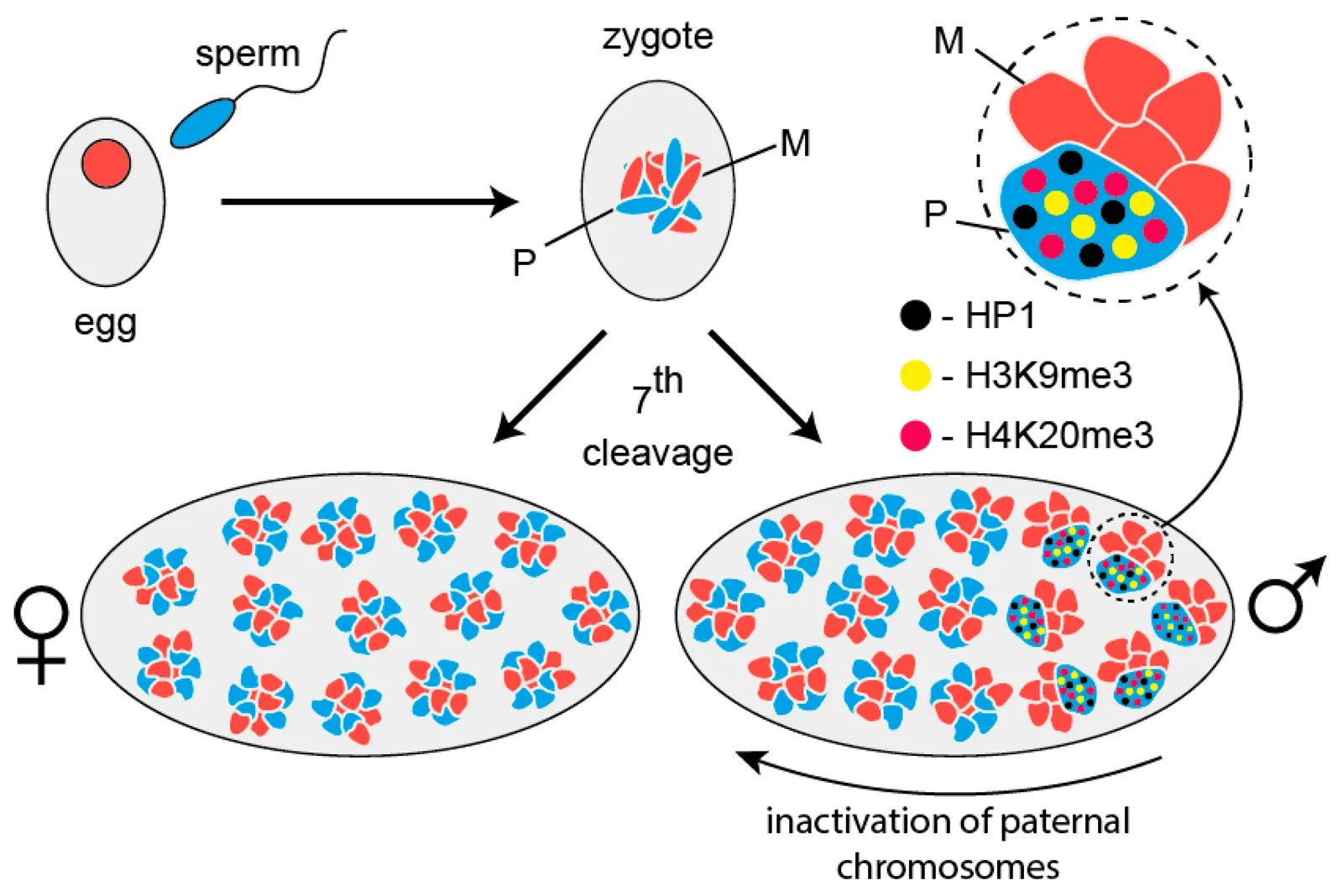
**Heterochromatinization of the paternal chromosome set in coccids.** Following fertilization, the sperm nucleus (blue) and egg nucleus (red) give rise to a diploid zygote containing ten chromosomes (2n = 10), which is the typical chromosome number in coccid species. In male embryos, heterochromatinization of the paternal chromosome set begins at approximately the seventh cleavage division and initially appears at one pole of the embryo (illustrated at the right-hand pole). Progressive condensation results in the formation of a chromocenter, representing the aggregated paternal chromosome set, shown at higher magnification in the inset. The chromocenter is enriched in the heterochromatin-associated histone modifications H3K9me3 (yellow) and H4K20me3 (magenta), together with the non-histone chromosomal protein HP1 (black). This process rapidly spreads as a wave to all nuclei in the male embryo. In female embryos, no chromocenter is formed, and the maternal and paternal chromosome sets remain indistinguishable throughout embryonic development. The colored dots indicate enrichment of H3K9me3, H4K20me3, and HP1 within the heterochromatic chromosome set; these signals largely overlap in the chromocenter of the male embryos after the inactivation of the paternal chromosome set [76,79,80]. Adapted from [6].

Chromosomal imprinting in mealybugs has long been regarded as a classic epigenetic puzzle that has attracted the attention of biologists for many years (reviewed in [3,74]). Following fertilization, both parental chromosome sets are euchromatic throughout the first five cleavage divisions, which proceed under maternal control. Around the sixth to seventh cleavage division—when transcription from the zygotic genome is first detected [75] and nuclei start migrating to the egg periphery—heterochromatinization of the paternal chromosome set occurs in male embryos, appearing as a “wave” that spreads from one end of the embryo to the other [76,77] (Figure 3). Early studies showed that this heterochromatinization leads to gene silencing: the paternal chromosome set in male embryos is transcriptionally inactive, whereas the maternal homologues within the same nucleus remain active [78].

**Figure 4:**
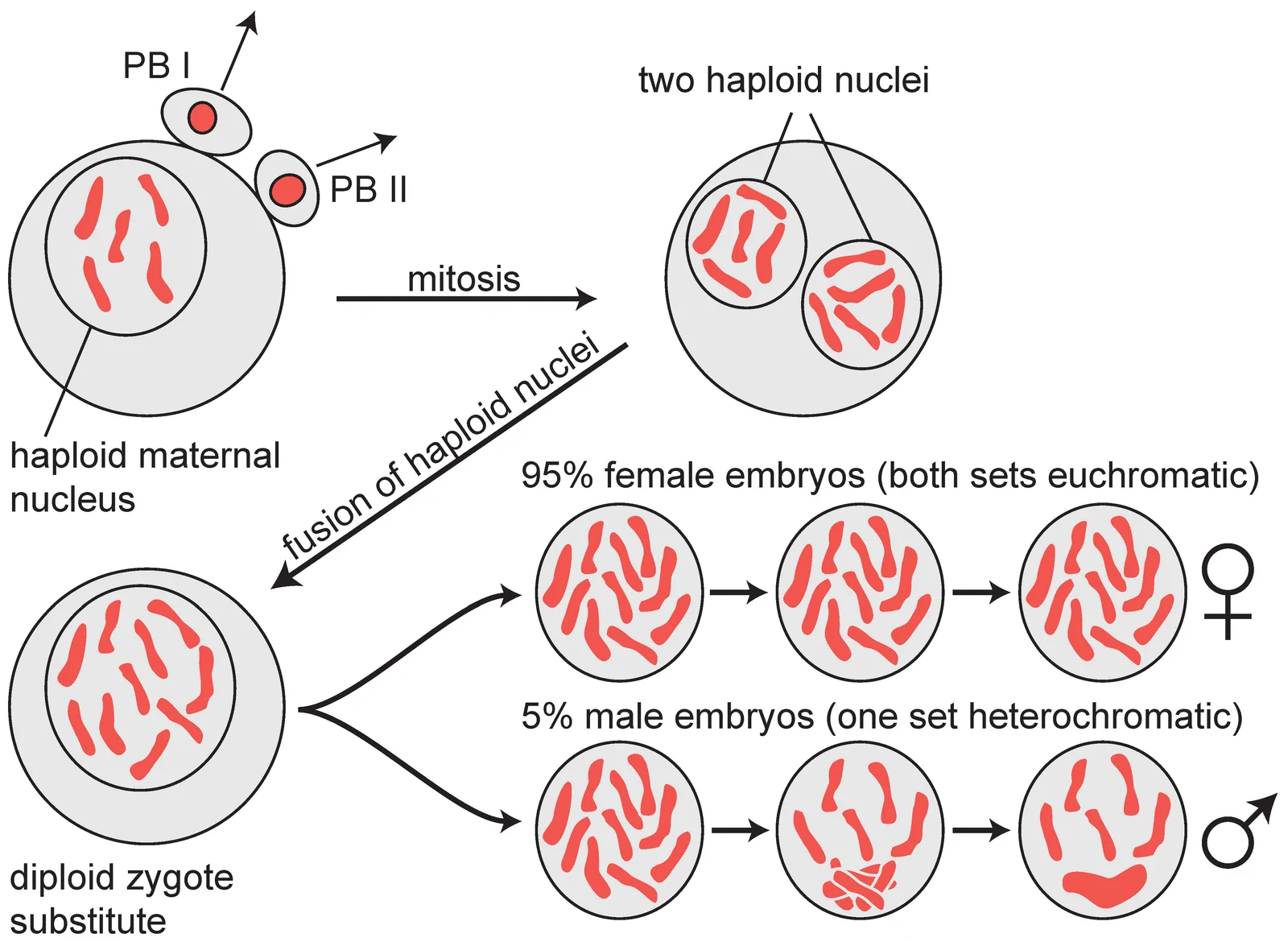
**Development of parthenogenetic male and female embryos in** ***Pulvinaria hydrangeae*****.** During meiosis, the maternal genome is reduced to a haploid nucleus containing five chromosomes, shown in red to indicate their exclusively maternal origin. After extrusion, polar bodies I and II do not participate in subsequent development. The haploid maternal nucleus undergoes a mitotic division, and the two daughter nuclei subsequently fuse to form a diploid zygote substitute. Approximately 95% of these embryos develop as females, in which both chromosome sets remain euchromatic. The remaining ~5% develop as males, where one chromosome set undergoes heterochromatinization. Because male embryos arise without any paternal genetic contribution, the imprint responsible for heterochromatinization must be established under maternal control through conditioning of the maternal ooplasm. Adapted from [6].

The question of whether sperm introduces an imprint into the egg that later directs heterochromatinization was addressed using the parthenogenetic soft scale insect evolutionarily related to mealybugs, *Pulvinaria hydrangea* [81]. In this species, embryos displaying either heterochromatinization or its absence arise parthenogenetically [81,82,83]. Female meiosis proceeds normally, producing typical haploid products. Notably, in *Pulvinaria hydrangea*, the egg nucleus undergoes a single mitotic division, after which two resulting haploid nuclei substitute the pronuclei and fuse to form a diploid zygote (Figure 4). The majority of embryos arising from this union are female and both their chromosome sets are euchromatic. However, toward the end of the egg-laying cycle, males are produced with one euchromatic and one heterochromatic chromosome set (Figure 4). It appears that parthenogenetic males are rudimental, as females have never been found with sperm in their ovarian ducts [81]. This phenomenon observed in the parthenogenetic species probably was derived from sexually reproducing ancestors, as it is unlikely that such a system evolved specifically to produce non-functional males [84]. Thus, in *Pulvinaria hydrangea* the heterochromatinization of one of the chromosome sets leading to the male organism development occurs without any paternal contribution (Figure 4).

This unusual example implies that one of the mitotically derived haploid nuclei is imprinted in the ooplasm before fusion and thus preprogrammed for inactivation. By similarity, it could be suggested that in sexually reproducing mealybugs, sperm chromosomes are, in some cases, imprinted in the ooplasm at the pronuclear stage and this subsequently directs their heterochromatinization and development of male; in other cases no imprint is placed, both chromosome sets remain euchromatic, resulting in female development.

Importantly, the egg nucleus is immune to imprinting activity in the ooplasm—since maternal chromosomes are always euchromatic. It was proposed that the egg contains a spatially restricted compartment capable of imprinting the sperm nucleus if it becomes positioned within this region. [84]. Accordingly, once chromosomes within a pronucleus (in sexually reproducing species) or the products of haploid mitosis (in parthenogenetic *Pulvinaria hydrangea*) are exposed to this imprinting environment, they become imprinted and are later subject to heterochromatinization. Alternatively, it could be suggested that eggs laid early do not imprint the paternal genome and therefore give rise to females, whereas eggs produced later acquire imprinting activity and develop into males. In this model, the restriction is temporal rather than spatial.

The nature of the imprint in mealybugs remains largely unknown. Although DNA methylation has been detected in *Planococcus citri* [85,86], its contribution to the imprinting process has yet to be established. *P. citri* has only the *DNMT1* gene, which has long been considered responsible for the maintenance of DNA methylation but recently been shown to also be involved in *de novo* methylation in some systems [87,88]. Indirect data suggest that there may be differential methylation between the two chromosomal sets in males [85]. RNAi-mediated depletion of *DNMT1* in another mealybug species, *Phenacoccus solenopsis*, caused some phenotypic effects, although their relationship to imprinting is uncertain [89]. RNAi-mediated depletion of *DNMT1* in *P. citri* embryos did not affect the proportion of embryos with an inactivated chromosome set (S. N. Belyakin, unpublished data). Thus, the involvement of DNA methylation in this mechanism in *P. citri* requires further investigation.

The downstream events leading to the inactivation of paternal chromosomes in mealybug males were investigated to some extent. The heterochromatic chromosome set becomes enriched in the epigenetic marks H3K9me3 and H4K20me3, together with the HP1-like protein Pchet2 [76,79,80,90]. The involvement of the H3K9me3:HP1:H4K20me3 pathway in heterochromatin formation has been tested experimentally. Knockdown of *Pchet2* by dsRNA results in pronounced decondensation of the heterochromatic set and the disappearance of H3K20me3, whereas H3K9me3 remains localized to residual heterochromatic blocks [76]. These findings demonstrate that heterochromatin is required for regulation of the transcriptionally inert paternal chromosome set.

Furthermore, using the RNAi approach it was shown that H3K9 and H4K20-specific HMTases are essential for this imprinted heterochromatinization. The strongest effect was observed for the *P. citri* homolog of H4K20-specific HMTase, as well as one of two homologs of *SetDB1* gene and one of two homologs of *Su(var)3-9* gene [91]. Together with previous data [76], this indicates that H3K9me3:HP1:H4K20me3 pathway plays important effector role in imprint reading and propagation in *Planococcus citri*.

### 3.2. Sciara (Bradisia) coprophila (Fungus Gnat)

Chromosomal imprinting in *Sciara coprophila* is more complicated: it involves the elimination of the entire paternal chromosome set in primary spermatocytes and the programmed elimination of paternal X chromosomes in the soma and germ-line (for reviews see [92,93,94]).

In *S. coprophila* males the entire paternal set of chromosomes is specifically eliminated during meiosis I [92,93,95]. Consequently, only the chromosomes of maternal origin proceed to the next stage and are finally packed into the sperm; these chromosomes (now paternal) will be transmitted to the next generation (for review see [94]).

During meiosis II, non-disjunction of the replicated maternal X chromosome (X_m_) occurs. Both copies make their way precociously to the monopole in secondary spermatocytes. The autosomes separate normally, so one set proceeds to the sperm while another set missing the X chromosome is lost. The final chromosomal composition of the sperm consists of X_m_ dyad and one set of autosomes (XXA) (for review see [94]).

Meiosis in females proceeds normally and results in haploid eggs (XA). Therefore, after fertilization, the zygote possesses a characteristic constitution in the nucleus with two sets of autosomes (maternal A_m_ and paternal A_p_) and three X chromosomes (two paternal X_p_s from the sperm and one maternal X_m_ from the egg).

**Figure 5:**
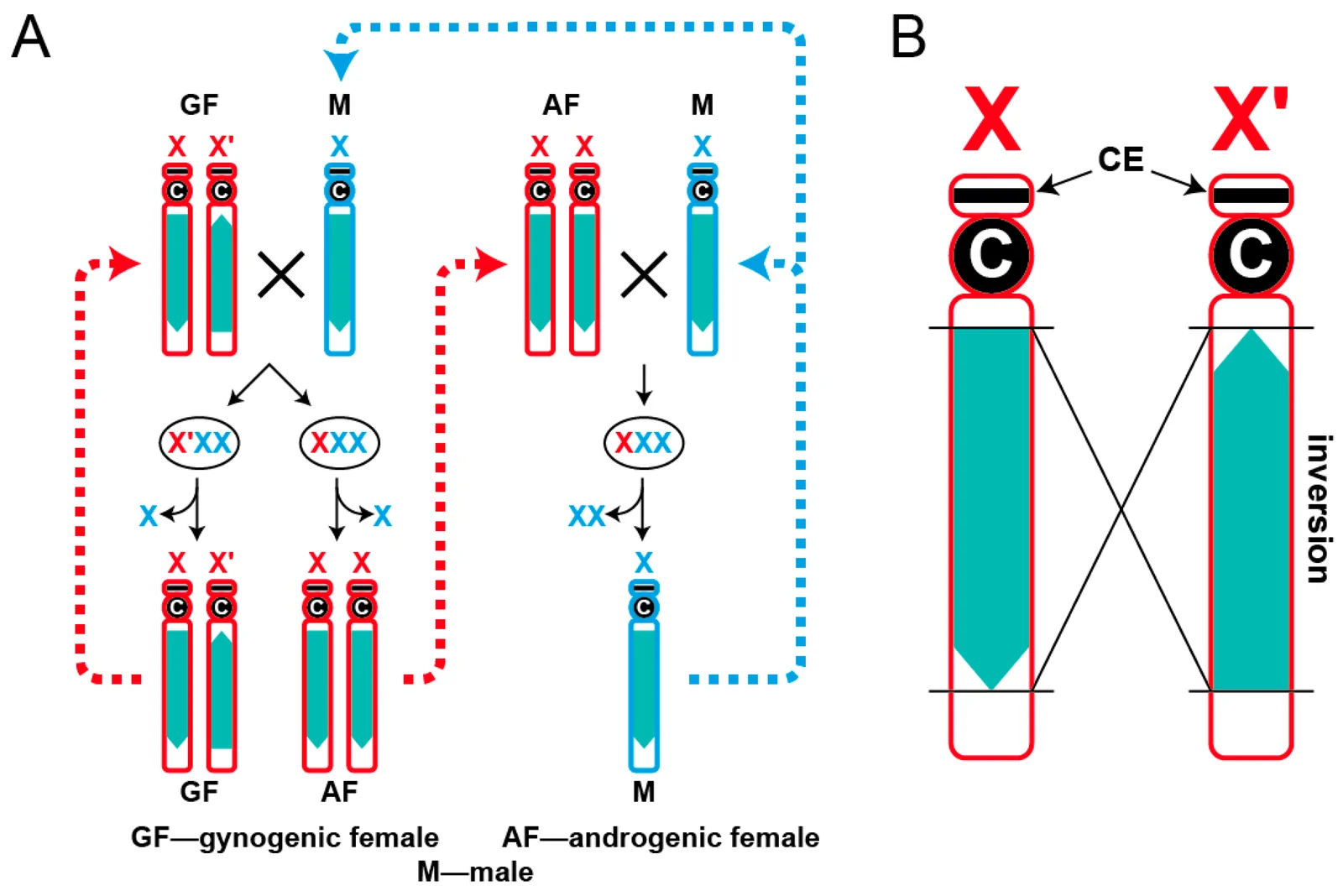
**Imprinting of X chromosomes in** *Sciara coprophila***.** (**A**) Inheritance and behavior of the X chromosomes during the *S. coprophila* life cycle. Chromosomes of maternal origin (X_m_ and X′_m_) are shown in red, whereas paternally inherited X chromosomes (X_p_) are shown in blue. Gynogenic females possess the XX′ genotype. Following fertilization, the zygote contains three sex chromosomes (X_p_X_p_X_m_ or X_p_X_p_X′_m_). During early embryogenesis, one of the two paternal X chromosomes is selectively eliminated, resulting in female progeny, including both gynogenic (XX′) and androgenic (XX) females. In contrast, androgenic females, which lack the X′ chromosome, produce eggs that are predetermined to eliminate both paternal X chromosomes after fertilization. Consequently, these females give rise exclusively to male offspring carrying a single X chromosome. (**B**) Schematic representation of the X and X′ chromosomes. The X′ chromosome carries a large paracentric inversion (green). The centromere is indicated by C, and the position of the Controlling Element (CE) on the short arm is marked by arrows. Adapted from [99].

This chromosome composition is maintained through the first few rounds of cleavage divisions until, during the 7th–8th divisions, paternal X chromosomes (X_p_) are eliminated in the somatic cells [92,93,95]. If two X_p_s are eliminated, the embryo develops as a male, whilst the elimination if one X_p_ results in female development (Figure 5A). The final somatic constitution is X_m_O in male and X_m_X_p_ in female [93]. The elimination of paternal X chromosomes in somatic cells occurs because of their non-disjunction during the anaphase, which prevents them from reaching the poles and ultimately leads to their loss [96].

The germ-line of both sexes is X_m_X_p_ because one X_p_ is eliminated from resting germ cells on the first day of larval life. The mechanism of this elimination seems to be completely different—it looks like one X_p_ chromosome is excluded from the intact nucleus through the nuclear envelope and consequently degenerates in cytoplasm [97]. The mechanism for germ-line elimination of X_p_ is not known.

The number of X_p_ chromosomes that will be eliminated in the somatic cells is directed by the ooplasm. In *Sciara coprophila* there are two types of females (Figure 5A). One type—gynogenic females—produces eggs that are preprogrammed to eliminate one of two X_p_ chromosomes after fertilization; all their progeny are X_m_X_p_ females. Accordingly, the eggs from androgenic females will eliminate both X_p_ chromosomes; these females only produce X_m_O males [93].

The difference between the androgenic and gynogenic females is the presence or absence of the X′ chromosome bearing a long para-centric inversion that was recently mapped using Hi-C approach [98,99] (Figure 5B). The X′ chromosome is inherited from mother to daughter and is found only in gynogenic females that always have XX′ composition (X′X′ females never appear). Androgenic females only bear two non-inverted X chromosomes (XX composition) [100]. Thus, the presence of X′ chromosome in the mother conditions her eggs to eliminate only one of two X_p_ chromosomes.

Elimination of the X_p_ chromosomes is regulated by a so-called “Controlling Element” (CE) on the X-chromosome that was mapped to heterochromomere II (H2) of polytene chromosome X, between the cluster of rDNA genes and the centromere [100,101,102] (Figure 5B). The existence of the CE was elegantly shown using the set of X to autosome translocations in classical early studies. Accordingly, the presence of paternally inherited H2 heterochromomere effectively drives the elimination of the translocated chromosome [1,101]. The CE not only regulates the elimination of X_p_ in the soma and germ line but also the non-disjunction of the X_m_ during meiosis II and precocious movement of the resultant X_m_ dyad to the monopole. Thus, imprinted X chromosome behavior completely depends on the CE.

Some hints about CE function were provided in the careful cytological study of the behavior of X_p_s during the 7th–8th embryonic cleavages [96]. The CE does not prevent the spindle attachment to the X centromere during the elimination. However, the X_p_ arms are unable to separate at anaphase, which ultimately leads to their loss. Thus, it seems that the CE acts *in* *cis*, but distantly, binding the replicated arms of the X_p_ chromosome. One of the possible interpretations for this behavior could be involvement of the presumptive ncRNA (lncRNA) that is expressed from the CE and spreads along the X_p_ chromosome arms that prevents their segregation in anaphase.

**Figure 6:**
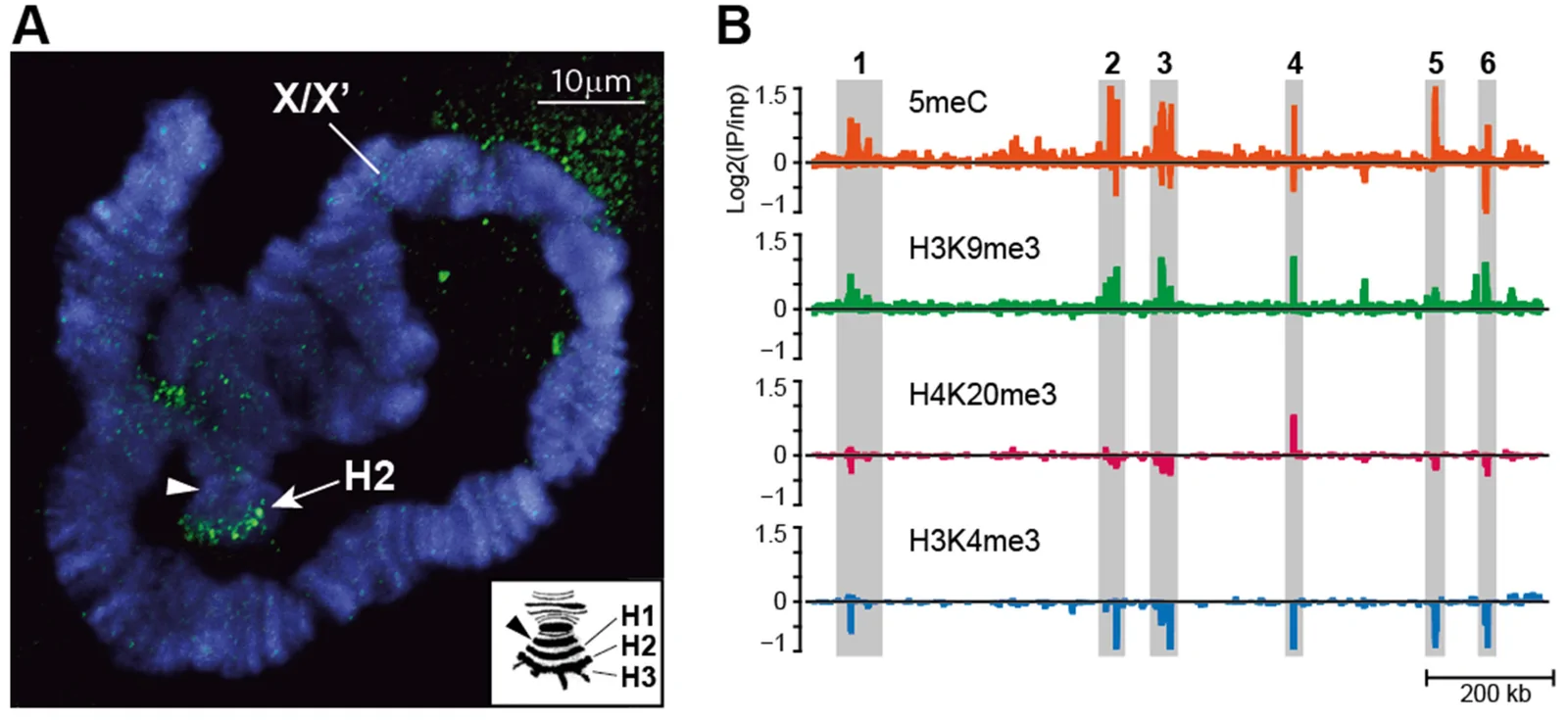
**5meC DNA methylation in chromosomes of** *Sciara coprophila*. (**A**) Polytene X and X′ chromosomes from a fourth-instar (L4) female larva are shown. Immunostaining with a 5-methylcytosine (5meC)-specific antibody (FITC, green) reveals a distinct enrichment of DNA methylation within the H2 heterochromomere containing the Controlling Element (CE, arrow). No comparable accumulation of 5meC is detected elsewhere on the chromosomes. DNA is counterstained with DAPI (blue). The arrowhead in the micrograph and the enlarged inset indicate the position of the centromere. Inset: schematic diagram of the H1, H2, and H3 heterochromomeres located on the short arm of the *S. coprophila* X chromosome (adapted from [103]). (**B**) High-resolution profiles of 5meC, H3K9me3, H4K20me3, and H3K4me3 in *S. coprophila* embryos across the 1.2 Mb region containing the CE. The 1.2 Mb genomic region mapped to the H2 heterochromomere is shown at higher resolution. Six discrete 5meC-enriched regions (peaks 1–6) are detected and coincide with corresponding H3K9me3 peaks. All six regions are depleted in the active histone modification H3K4me3. In contrast, H4K20me3 exhibits only limited overlap with the repressive chromatin marks, with the exception of peak 4. Profiles are displayed as log_2_(IP/Input) values, where positive values indicate enrichment of the corresponding epigenetic mark and negative values indicate depletion. Adapted from [99].

The exact position of the CE is not known yet; however, a recent study has demonstrated that in post-elimination embryos the H2 of the remaining X chromosomes contain a few adjacent clusters of 5meC DNA methylation coinciding with the enrichment with H3K9me3 histone mark and depleted in H3K4me3 marks (Figure 6). No comparable DNA methylation was found in the remaining genome, making one or both of these clusters the best candidates for the CE role existing to date [99].

The molecular mechanism of imprinting in *Sciara coprophila* is not yet fully understood. Since the number of the X_p_ chromosomes to be eliminated fully depends on the cytoplasm of the egg, it seems that the epigenetic state of the paternal CE cannot be decisive in this process [84]. In turn, the presence of DNA methylation in the remaining X chromosomes after elimination suggests that the CE on the X_m_ is pre-methylated by the mother and, in the case of females, one of the X_p_ chromosomes also acquires this mark, while another one does not and is subsequently eliminated in somatic cells.

**Figure 7:**
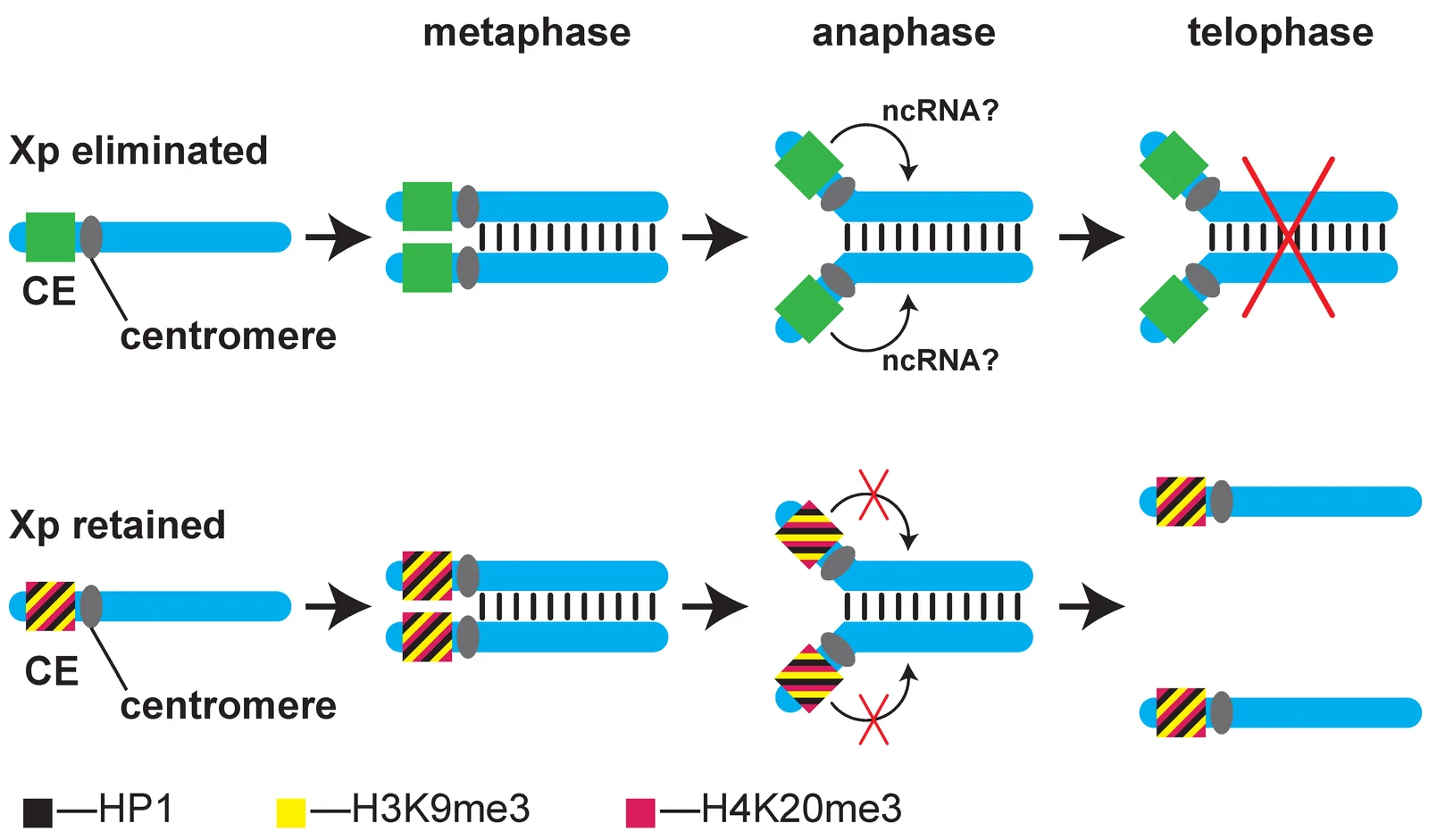
**Model for the elimination of paternal X chromosomes in the embryonic soma in** *Sciara coprophila***.** Top row: The Controlling Element (CE) on the paternal X chromosome destined for elimination remains transcriptionally active. Following DNA replication, sister chromatids align normally at metaphase. During anaphase, centromere separation is initiated, but expression of the CE gives rise to a putative non-coding RNA that acts *in cis* to prevent separation of the sister chromatid arms. Consequently, the chromatids remain connected at the metaphase plate and are ultimately eliminated. Bottom row: In embryos derived from eggs produced by gynogenic XX′ females, one paternal X chromosome is retained. In this case, the CE is embedded within heterochromatin and the putative ncRNA is not expressed. Sister chromatids align normally at metaphase, and both the centromeres and chromosome arms separate during anaphase, allowing each chromatid to segregate into a daughter nucleus. Adapted and modified from [5,6].

The presence of repressive histone mark H3K9me3 and to some extent H4K20me3 at the presumptive CE [6,99] may be a part of mechanism that propagates the epigenetic state of the CE though the cell divisions, ensuring the normal behavior of the X chromosomes in mitoses (Figure 7). It could be suggested that, in the egg from the XX′ gynogenic mother, the expression of the putative ncRNA from the CE is prevented on the single remaining X_p_ chromosome by the H3K9me3:HP1:H4K20me3 pathway that assembles a repressive chromatin state. This state is then inherited in cell generations maintaining the proper number of chromosomes in the body. This hypothesis adopts the regulatory principles of mammalian imprinting and could be tested experimentally.

In turn, in the other X_p_ chromosome that is lost in 7th–8th embryonic cleavages, the CE, remains active, which leads the ncRNA production which induces the mechanism of the elimination. In this model, in eggs from XX androgenic mothers both paternal CEs are expressible and, subsequently, both X_p_ chromosomes are eliminated. The maternal CE would be inactivated during oogenesis (Figure 7).

As mentioned above, the specific elimination of the entire paternal chromosome set occurs during meiosis I in males. This selectivity implies that all paternal chromosomes (X_p_ and A_p_) are marked and maintain this mark through several cell divisions in the germ line cells. The nature of this mark remains unknown.

The relative contribution of the germ-line and post-fertilization mechanisms to the imprinting phenomena in *Sciara* appears to have been resolved in favor of the egg as being the site of imprinting. However, given advances in molecular techniques, it may be worth investigating that brief window in secondary spermatocytes when the chromosomes inherited from the mother become marked as paternal before packing in the sperm. That is, there might be two imprinting systems working in parallel, one for the elimination of the paternal chromosome set in primary spermatocytes and another for elimination of X_p_s from the soma and germ line. Furthermore, the recently identified presumptive CE region (Figure 6B) should be functionally validated in a transgenic system to check if it is capable of causing chromosome elimination in a transgenic system. The molecular mechanism of CE function and the presumptive role of ncRNA represent another fascinating question to be addressed.

## 4. Proto-Imprinting in Nematode *Caenorhabditis tropicalis*

An intriguing case of a parent-of-origin effect with elements of imprinting was found in the nematode *Caenorhabditis tropicalis*. In this species, females are hermaphrodites and can produce progeny by selfing. Males also exist and participate in sexual reproduction.

Nematodes are among the rare examples of animals that have a toxin–antidote (TA) system (another example is the flour beetle *Tribolium castaneum* [104]). The TA system in nematodes consists of two functional parts that are closely linked in the genome and are inherited as a single selfish unit. One part encodes the toxin that is maternally loaded to the egg. Another part produces the antidote that is expressed zygotically, allowing normal development. This mechanism favors the propagation of the TA system in the population [105,106].

**Figure 8:**
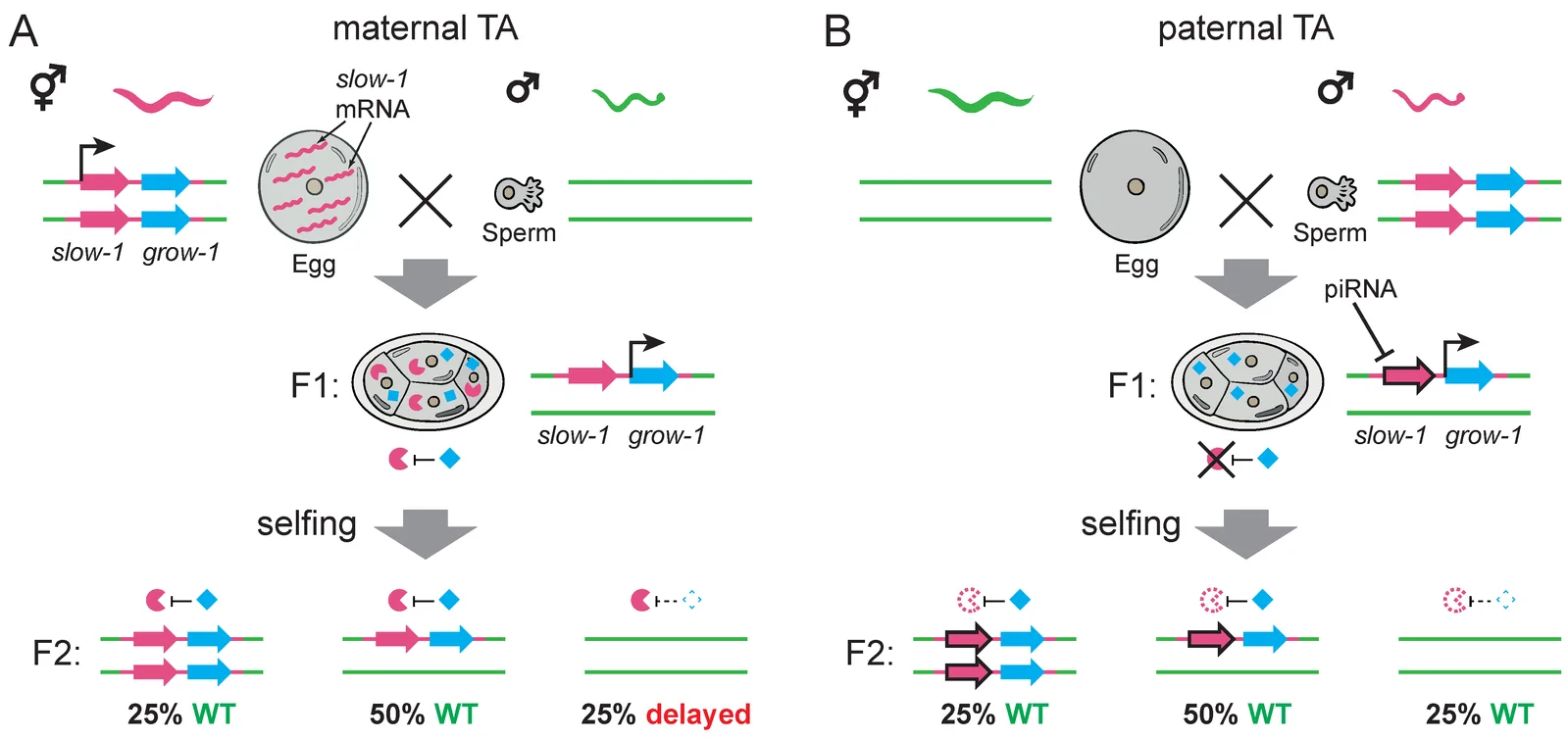
**Parent-of-origin effect on the selfish genetic element in** *Caenorhabditis tropicalis***.** Expression of the toxin-antidote (TA) system *slow-1/grow-1* depends on its parental origin. (**A**) In F1 heterozygous females, when the TA element is inherited maternally, the egg is loaded with *slow-1* toxin mRNA. Following fertilization, the zygotically expressed *grow-1* antidote gene enables normal development. As a result of self-fertilization, three classes of F2 progeny are produced according to Mendelian segregation. Homozygotes carrying the TA element (25%) and heterozygotes (50%) develop normally. In contrast, F2 progeny lacking the TA element are unable to produce the antidote and exhibit a pronounced developmental delay. (**B**) In the reciprocal cross, when the TA element is inherited paternally, *slow-1* expression is suppressed by the piRNA pathway in F1. Consequently, following self-fertilization, the amount of toxin inherited by the F2 progeny is greatly reduced, allowing all three genotypic classes of the next generation to develop normally [2].

In the previously characterized systems in nematodes the parental origin of the TA locus did not affect the rescue of the progeny [107]. However, a recent study has shown that, in the case of one of the TA systems (*slow-1/grow-1*) in *Caenorhabditis tropicalis*, parental origin makes a difference (Figure 8). If the heterozygous female inherits the *slow-1/grow-1* locus from her mother, it is transcriptionally active in the germ line and all the eggs are filled with the *slow-1* mRNA encoding the toxin. After the self-fertilization, all the eggs missing the TA (25%, according to the Mendelian distribution) demonstrate severe developmental delay (Figure 8A). However, if the female inherits this TA locus from its father, its expression in the germline is considerably suppressed, resulting in a lower dosage of the toxin loaded to the egg and, consequently, to a reproducibly low percentage of delayed progeny after self-fertilization [2] (Figure 8B). This peculiar pattern is not observed in other TA systems [104,108].

Details of the mechanism ensuring the repression of the paternally inherited *slow-1/grow-1* in the female germline have not yet been fully described. However, it was shown that piRNAs are involved in this process [2] (Figure 8B). Whether the H3K9me3 histone modification and HP1 homologs are involved remains to be determined. It seems reasonable to suggest this option, as in other organisms piRNA-mediated repression is usually associated with HP1-driven repression [109]. However, the involvement of other repressive epigenetic mechanisms cannot be excluded.

The *slow-1/grow-1* TA system combines the features genomic imprinting with classic hybrid dysgenesis systems. As such, the *slow-1/grow-1* TA system could represent an intermediate stage in the evolution of genomic imprinting.

## 5. Conserved Core Mechanism of Genomic Imprinting

Examples from very distinct taxa described above illustrate two important characteristics that likely reflect the evolution of genomic imprinting. First, genomic imprinting arose independently multiple times during evolution. Indeed, there is no direct evolutionarily consistent progression between the imprinted loci in mammals, X_p_ chromosome elimination in *Sciara coprophila*, and inactivationof the entire chromosomal set in *Planococcus citri*. Moreover, examples of non-canonical imprinting systems (reviewed in [57]) and imprinting in plants (see [7,110,111,112]) suggest that various epigenetic mechanisms can be adapted to distinguish genetic material of different parental origin. The proposed imprinting-like system in *Caenorhabditis tropicalis* illustrates how genomic imprinting can evolve by co-opting pre-existing epigenetic regulatory mechanisms.

Second, the examples above show that common components are shared between different canonical imprinting mechanisms (Table 1). These include H3K9 methylation and HP1/HP1-like proteins. Such chromatin determinants are characteristic of heterochromatin and are highly conserved from yeast to mammals [113]. This epigenetic repressive mechanism is essential for imprinting.

Studies in mammalian systems have shown that imprinted lncRNAs are known to regulate parental-specific expression of neighboring imprinted genes [114]. The *Rsx* ncRNA is involved in imprinted inactivation of the paternal X-chromosome in marsupials. In *S. coprophila*, imprinted elimination participation of the ncRNA is strongly suggested, however was not shown yet. In the nematode *slow-1/grow-1* TA system, piRNAs could be considered to be performing a similar function. Whether ncRNAs are involved in the imprinting mechanism in *P. citri* is completely unclear (Table 1).

Another component that is well established as being involved in genomic imprinting in mammals is DNA methylation, where DNA methylation marks imprinted loci in the gametes, which is retained through the genome-wide DNA demethylation events that take place during pre-implantation embryogenesis (Table 1). Maintenance of the methylation mark requires the H3K9me3:HP1:H4K20me3 pathway. Enrichment of DNA methylation at the region of the CE location in *S. coprophila* is associated with the H3K9me3:HP1:H4K20me3 pathway. In the coccid, *P. citri*, a role for DNA methylation imprinting is less well characterized but again, there is strong evidence for the H3K9me3:HP1:H4K20me3 pathway in regulating imprinting in coccids.

Interestingly, an alternative, non-canonical mechanism of imprinting relies on the H3K27me3 as a primary mark (for a review, see [115]). Histones are absent from sperm and are replaced by protamines; therefore, H3K27me3-mediated imprint is inherited maternally. It should be noted that this type of imprinting may be transient, because allele-specific H3K27me3 deposition often fades over successive cell generations [116]. However, there are also examples in which non-canonical imprinting persists through multiple cell generations. For example, it has been shown that when DNA methylation is acquired *de novo* at the non-canonically imprinted loci it can serve as a secondary epigenetic mark that maintains parent-of-origin-dependent regulation of genes [117,118]. These examples suggest that, even in non-canonically imprinted systems, DNA methylation may play an important role; however, rather than being inherited from the parents, it is established after fertilization to maintain the imprinted state.

Altogether, genomic imprinting appears to have arisen convergently in different taxa and to have been adapted for diverse biological functions. The existence of parent-of-origin effects, therefore, should not be considered solely within the narrow context of imprinted genes in mammals. Notably, the participation of a conserved set of molecular components in imprinting mechanisms across diverse organisms suggests that the ancient H3K9me3:HP1:H4K20me3 epigenetic pathway constituted an evolutionarily reusable module that was repeatedly co-opted to distinguish maternal and paternal genomes.

## Figures and Tables

**Table 1:** The main components of imprinting in the described biological models.

	5meC	H3K9me3/HP1	H4K20me3	ncRNA/piRNA
***Mus musculus***	+	+	+	+
**Marsupials**	+	+	+	+
***Planococcus citri***	?	+	+	?
***Sciara coprophila***	+	+	+	?
***Caenorhabditis tropicalis***	–	+	?	+

“+”—involved, “–”—apparently, not involved, “?”—suggested/not enough data.

## Data Availability

No new data were created or analyzed in this study. Data sharing is not applicable to this article.
